# Recent Advances in Design and Fabrication of Nanocomposites for Electromagnetic Wave Shielding and Absorbing

**DOI:** 10.3390/ma14154148

**Published:** 2021-07-26

**Authors:** Yang Huang, Ming Chen, Aming Xie, Yu Wang, Xiao Xu

**Affiliations:** 1 Jiangsu Co-Innovation Center of Efficient Processing and Utilization of Forest Resources and International Innovation Center for Forest Chemicals and Materials, Nanjing Forestry University, Nanjing 210037, China; asd1738374911@yeah.net (M.C.); wy11051617@163.com (Y.W.); xuxiao202102@163.com (X.X.); 2 School of Mechanical Engineering, Nanjing University of Science and Technology, Nanjing 210094, China

**Keywords:** dielectric loss, magnetic loss, electromagnetic pollution, absorption mechanisms, heterostructures

## Abstract

Electromagnetic (EM) pollution has raised significant concerns to human health with the rapid development of electronic devices and wireless information technologies, and created adverse effects on the normal operation of the sensitive electronic apparatus. Notably, the EM absorbers with either dielectric loss or magnetic loss can hardly perform efficient absorption, which thereby limits their applications in the coming 5G era. In such a context, the hotspot materials reported recently, such as graphene, MXenes, and metal-organic frameworks (MOF)-derived materials, etc., have been explored and applied as EM absorbing and shielding materials owing to their tunable heterostructures, as well as the facile incorporation of both dielectric and magnetic components. In this review, we deliver a comprehensive literature survey according to the types of EM absorbing and shielding materials, and interpret the connectivity and regularity among them on the basis of absorbing mechanisms and microstructures. Finally, the challenges and the future prospects of the EM dissipating materials are also discussed accordingly.

## 1. Introduction

In recent years, owing to the rapid development of communication technology and the extensive utilization of electronic devices, such as telecommunication, local area network and radar systems, electromagnetic (EM) waves have been generated in various frequency bands in our modern society. Serious EM radiation has aroused intensive concerns as these EM interferences (EMI) not only threaten the physiological functions of human beings but also disturb the normal operation of sensitive electronic apparatuses. It is of significant importance to dissipate the EM waves to ensure the safety of the operators and the normal function of the sensitive systems. High radiofrequency interference (RFI) is ubiquitous in some areas, where industrial microwave ovens or mobile communication devices with high transceivers are widely used [1,2]. Moreover, EM radiation is frequently generated by radio/TV and radar. Currently, the EM pollution has generated from popular portable electronic devices with high power, leading to the disorder of neighboring electronic equipment [3]. Therefore, it is imperative to develop high performance EM shielding and absorbing materials that are envisioned and can be employed in areas of commercial/personal communication, space explorations, or electronic medical devices [4,5,6].

**Figure 1:**
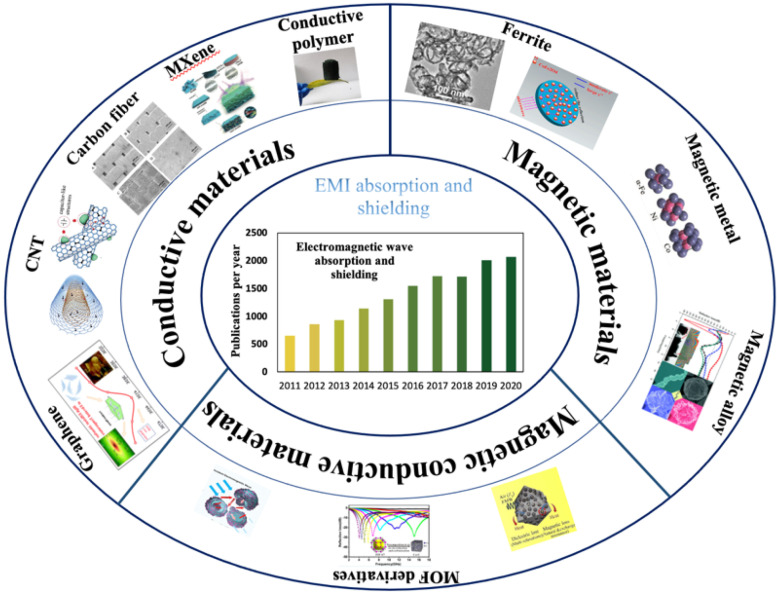
Materials applied in EMI absorption and shielding and the relevant publication numbers in the Web of Science.

In the past decade, much attention has been paid to the research related to EM wave absorbing and shielding. As shown in Figure 1, various types of conductive materials (including carbon nanotube, carbon fiber, graphene, and MXene, etc.) and magnetic materials (Fe, Co, Ni, ferrite, and alloys, etc.) have been successfully utilized in the dissipation of EM wave through dielectric loss or magnetic loss [7,8,9,10,11,12,13,14,15,16,17,18,19].

Generally, conductive and magnetic materials are applicable in the field of the EMI shielding due to their outstanding dielectric and magnetic properties. Dielectric loss materials, such as carbon material, MXene, SiC, and conductive polymers possess decent electronic conductivity, but suffer from impedance mismatching and narrow effective absorption bandwidth. In contrast, magnetic responding materials perform good EM wave absorbing capacity with wide frequency bandwidth [20]. However, high gravimetric density and poor corrosion resistance limits their utilization. It is widely accepted that the judicious design of the microstructures, the configurations, and the heteroatomic dopants of the nanomaterials are believed to effectively block the propagation of the EM waves. In this review, we start with summarizing the shielding mechanism and then systematically highlight the recent developments of the EM wave absorbing materials. Effects of the properties and configuration of these materials on EM wave shielding and absorption are also comprehensively presented along with objective interpretation from scientific perspectives. Finally, the remaining challenges are commented, and the provident prospects for further development in this blossoming field are addressed.

## 2. Basic Principles of EMI Shielding

### 2.1. Shielding Mechanism

EMI shielding is the process used to block the propagation of EM wave by means of dissipation from conductive or magnetic mediums [21]. The attenuation capability of an EMI shielding material is defined as electromagnetic shielding effectiveness (SE), which is the ratio in decibels (dB) of the incident and transmitted energies or fields of the EM wave, and is given in Equations (1)–(3) [22]:

(1) $${\mathrm{SE}}_{P}=10\log(P_{\mathrm{in}}/P_{\mathrm{out}}),$$

(2) $${\mathrm{SE}}_{E}=20\log(E_{\mathrm{in}}/E_{\mathrm{out}}),$$

(3) $${\mathrm{SE}}_{H}=20\log(H_{\mathrm{in}}/H_{\mathrm{out}}),$$

where P, E, and H are the strength of plane wave (W), electric field (N C^−1^), and magnetic field (A m^−1^) of the EM wave, respectively. The subscripts “in” and “out” mean the strength of the incident and transmitted wave through the EM material.

**Figure 2:**
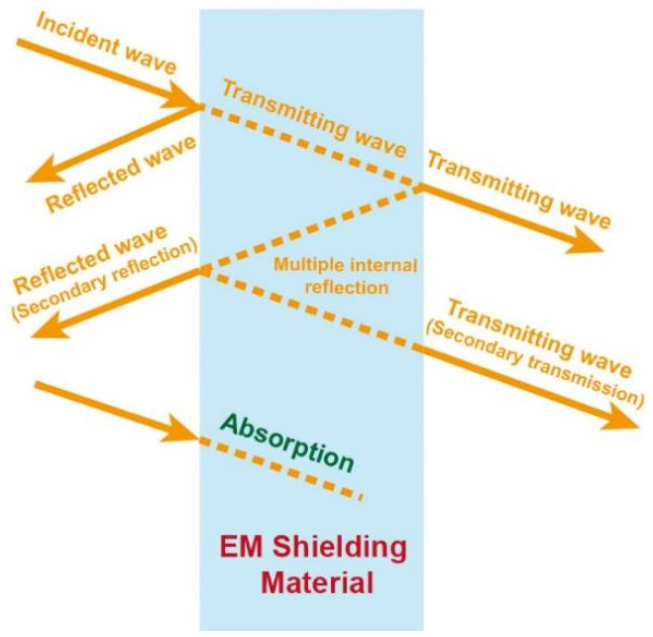
Schematic representation of the shielding mechanism.

When the EM wave strikes the shielding material, there will exist reflection loss (SE_R_), absorption loss (SE_A_), multiple reflection loss (SE_M_), and transmission loss at the same time [23], as shown in Figure 2. The concept of the reflection loss in the EMI shielding is the difference between the initial incident waves and the waves penetrating the shield. The total SE of an EMI shielding material (SE_T_) is the total of the three SE derived from SE_R_, SE_A_, and SE_M_ as depicted in Equation (4):

(4) $${\mathrm{SE}}_{T}={\mathrm{SE}}_{R}+{\mathrm{SE}}_{A}+{\mathrm{SE}}_{M},$$

in which if SE_A_ is less than 10 dB, SE_M_ can be neglected [24].

An electric field of the plane wave decreasing exponentially as it travels inside a conductive material. The distance at which the field strength drops to 1/e is defined as skin depth (δ), which is given by Equation (5):

(5) $$\delta ={\left(\sqrt{\pi f\mu \sigma }\right)}^{-1},$$

where f is wave frequency, µ and σ are the relative permeability and electrical conductivity of the shielding material, respectively. If the skin depth is lower than the thickness of the shield or any conductive particle inside, the influence of SE_M_ should also be ignored.

When an EM wave transmits onto the shield, there exists the possibilities of absorption, reflection, and transmission of the EM wave, whose coefficients are denoted by A, R, and T, respectively, indicating the responses of the shielding to the incident EM wave [25]. The reflective part of the EM wave is comprised of reflection from the shielding material surface and the secondary reflection in the internal of materials, which tends to happen on the high conductivity surface with a large number of charge carriers [26]. Inside of the material, a fraction of EM energy is converted to thermal energy [27,28]. From the scattering parameters (S_mn_) measured by a vector network analyzer (VNA) system, the absorption factor (A), transmission factor (T), and reflection factor (R) can be calculated from Equations (6)–(8) [29];

(6) $$R={\left|S_{11}\right|}^{2}={\left|S_{22}\right|}^{2},$$

(7) $$T={\left|S_{21}\right|}^{2}={\left|S_{12}\right|}^{2},$$

(8) A=1−R−T,

where “1” and “2” represent the network analyzer port receiving the EMI radiation and the port transmitting the incident EM wave.

The corresponding SE_R_, SE_A_, and SE_T_ can be expressed as Equations (9)–(11):

(9) $${\mathrm{SE}}_{R}=-\log_{10}\left(1-R\right),$$

(10) $${\mathrm{SE}}_{A}=-\log_{10}\left(\frac{T}{1-R}\right),$$

(11) $${\mathrm{SE}}_{T}={\mathrm{SE}}_{A}+{\mathrm{SE}}_{R}\;({\mathrm{SE}}_{M}\;\mathrm{is}\;\mathrm{negligible}).$$

### 2.2. EM Wave Absorption

Since the reflected wave will cause the secondary EM pollution, EMI shielding materials with outstanding absorption have been urgently pursued by researchers. As is well known, the EM wave materials can attenuate 90 and 99% electromagnetic waves if the reflection loss (RL) value is lower than −10 dB and −20 dB, respectively. The RL refers to the difference between the initial incident waves and the final reflected waves [30]. According to the transmission line theory, RL at the shield surface as a function of impedance is given by Equation (12) [31,32]:

(12) $$\mathrm{RL}\left(\mathrm{dB}\right)=20\log\left[\frac{Z_{in}-Z_{0}}{Z_{in}+Z_{0}}\right].$$

The *Z_in_* and *Z*_0_ can be defined as Equations (13) and (14):

(13) $$Z_{in}=\sqrt{\frac{\mu_{r}}{\varepsilon_{r}}\tan h\left[j\frac{2\pi fd}{c}\sqrt{\mu_{r}\varepsilon_{r}}\right]},$$

(14) $$Z_{0}=\sqrt{\frac{\mu_{0}}{\varepsilon_{0}}}.$$

where *Z_in_* is the input impedance of the microwave absorption layer at the surface; *Z*_0_ refers to the intrinsic impedance of the free space (about 377 Ω); *µ_r_* and *ε_r_* are the relative complex permeability and permittivity, respectively; *c* is the velocity of the light and *d* is the thickness of the absorber.

Generally, the electrical conductivity of the materials is dominated by the hopping electrons [33]. Electrons can migrate in a continuous carbon framework (migrating electrons), or leap across between the intra-layer defects and the inter-layer interface (hopping electrons). In addition, the increased temperature can activate the hopping electrons to jump the barriers, which enhances the conduction loss.

Typically, the dielectric properties of wave absorbers are also correlated with the electron, ion, dipolar. and interfacial polarization. Naturally, the electron and ion polarizations are omitted as they occur in THz and PHz frequency range. Thus, the polarization loss in GHz frequency range is mainly ascribed to dipolar and interfacial polarization. Dipole polarization is mainly due to the polarization relaxation of defects and functional groups under high-frequency alternating electric field. Consequently, ε′ and ε″ will decrease and produce a typical frequency dispersion behavior [34]. Interfacial polarization and the correlated relaxation are formed in the heterogeneous systems. The difference of the conductivity between the layers leads to the accumulation and uneven distribution of space charge, which results in the macro dipole moment to dissipate incident electromagnetic wave [8].

Magnetic loss mainly derives from magnetic hysteresis, eddy current losses, natural resonances, domain wall resonances, and exchange resonances [35]. However, the energy conversion in the microwave frequency range comes from the eddy current, natural resonances, and exchange resonances. Generally, natural resonances occur at a lower frequency (2–10 GHz) and exchange resonances appear at high frequency region (>10 GHz). When the values of µ″(µ′) f^−1^ remain constant as frequency changes, the eddy current will be the only contributor to the magnetic loss [36].

The attenuation constant (α), the EM absorption ability of an EMI shielding material, can be confirmed by Equation (15):

(15) $$\alpha =\frac{\sqrt{2}\pi f}{c}\times \sqrt{\left(\varepsilon \prime \prime \mu \prime \prime -\varepsilon^{\prime }\mu^{\prime }\right)+\sqrt{{\left(\varepsilon \prime \prime \mu \prime \prime -\varepsilon^{\prime }\mu^{\prime }\right)}^{2}+{\left(\varepsilon \prime \prime \mu \prime \prime +\varepsilon^{\prime }\mu^{\prime }\right)}^{2}}}.$$

The ε′ and µ′ refer to the real part of the permittivity and permeability, respectively, which represents the storage capacity of the electrical and magnetic energy. The ε″ and µ″, the imaginary part of permittivity and permeability, respectively, are related to the loss of electrical and magnetic energy. Furthermore, the dielectric dissipation factor (tan δ_ε_ = ε″/ε′) and magnetic dissipation factor (tan δ_µ_ = µ″/µ′) provide a measure of how much power is lost in material versus how much is stored. To be an ideal EMI shielding material according to those above equations, the material needs to meet the requirements on perfect impedance matching (|*Z_in_*/*Z*_0_| ≈ 1) and appropriated balance between dielectric dissipation and magnetic dissipation. When the value of |*Z_in_*/*Z*_0_| is close to 1, there are less electromagnetic waves reflecting on the surface of the absorber, while the majority is the incident inside the absorbing body. The thickness of the absorber also affects the intensity and the position of the minimum RL. According to the 1/4 wavelength theory, the relationship of the matching thickness t_m_ and the matching frequency f_m_ is expressed by the following Equation (16) [37]:

(16) $$t_{m}=n\lambda /4=\mathrm{nc}/\left(4f_{m}{\left(\left|\mu_{r}\right|\left|\varepsilon_{r}\right|\right)}^{1/2}\right),$$

where n = 1, 3, 5, …, λ is the incident wavelength of the electromagnetic wave. The reflected waves at the interface of the air and the absorber will offset what generates the interface of the absorber and the conductive backplane when t_m_ and f_m_ meet the above equation.

### 2.3. Relationship of the EMI Shielding and EM Absorption

**Figure 3:**
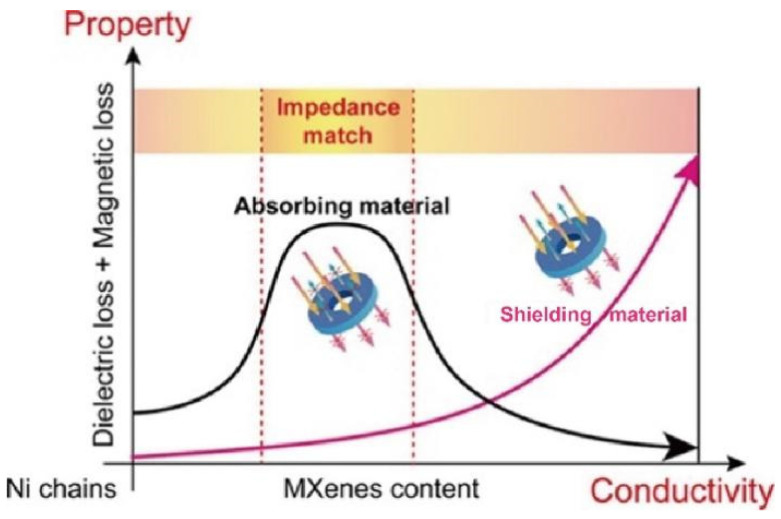
Relationship between impedance match and conductivity for the design of EM shielding and absorption materials.

Cao et al. [38] deeply investigated the dielectric relaxation and EM response of the MXene-based materials. The surface of the conductive MXene is filled with free electrons, leading to the incident EM wave to be reflected at the surface. As the electrical conductivity increases, the reflectivity elevates and the absorption rate decreases. Thus, MXene with the conductivity above the percolation threshold are more propitious to EMI shielding than EM absorption. Lv et al. [39] proposed a phase diagram of the relationship between the conductor/magnet components ratio and the EM shielding and absorption properties. As shown in Figure 3 the MXene/Ni composites with a moderate MXene content possess superior dielectric loss, magnetic loss, and impedance matching, which are suitable for using as an EM wave absorber. With the increment of the MXene contents, the enhanced conductivity favors to EMI shielding. Arjmand et al. [40] compared the EMI shielding properties of multi-walled carbon nanotube/polystyrene (MWCNT/PS) prepared by injection molded method and compression molded technique. It has been observed that the compression molded samples performed a better absorption capability and the same reflection capability compared with the injection molded samples. This is ascribed to the enhanced connectivity between the fillers in MWCNT/PS fabricated by compression molding. The enhanced connectivity increased the polarization of PS polymer layer between MWCNTs, which led to the lowering of the real permittivity and imaginary permittivity. From the above results, it can be seen that the absorption of the EM wave depends on the gap/connectivity of the filler, while EMI shielding materials are influenced by the ratio of conductive fillers.

## 3. Electromagnetic Functional Materials

### 3.1. Carbon Materials

Carbon materials such as carbon nanotube [41], graphene [42], and carbon nanofiber [43] are excellent materials for EMI shielding due to their lightweight, low-density, anti-corrosion, high electrical conductivity, and thermal stability [44,45], as shown in Table 1. The large specific surface area of these nanomaterials lowered their concentration in the paraffin matrix for the construction of an overall conductive network. In addition, the polarization centers derived from the defects and the groups on surface usually induce the dipolar relaxation loss. Moreover, carbon-based nanomaterials possess a high degree of graphitization with enhanced electrical conductivity, which will increase the imaginary part of the relative complex permittivity, and thereby contribute to superior conduction loss [46].

#### 3.1.1. 1D Nanomaterials

Among the various morphologies, one-dimensional (1D) nanostructures have attracted wide interests for their considerable advantages, such as low density and large aspect ratio [60], which performed excellent EMI shielding with absorption-dominated behavior [61]. Typically, materials with nanofibrous structure belong to 1D nanomaterials, including carbon nanotubes (CNTs) [62] and carbon nanofibers [63]. Apart from the nanostructure of carbon materials, the assembling configurations of these materials is also pivotal in case of the layered, cellular, or porous structure.

CNTs have unique electrical, mechanical, and thermal properties and therefore have recently been attracting much attention [8,48]. According to the layers of graphitic nanosheets, CNTs can be divided into CNT-single-walled (SWCNTs) and CNT-multi-walled (MWCNTs) [64]. The high aspect ratio, high electrical conductivity, and excellent mechanical properties of CNTs make them the perfect choice for fabricating conductive composites for EMI shielding [65]. In addition, the few defects of SWCNTs lead to higher conductivity compared to MWCNTs [47]. The large aspect ratio of CNTs facilitates them to form a conductive network in a polymer matrix. That is the reason why CNT has been considered as an excellent candidate for EMI shielding material [66]. Li et al. [67] and Huang et al. [68] proved that the EMI SE of long-SWCNTs composites was much higher than that of short-SWCNTs composites in the same loading ratio, benefiting from the lower percolation threshold (0.062%) with a high electrical conductivity (~0.14 S·m^−1^). The significant increase in EMI SE of annealed short-SWCNTs composites was attributed to partial removal of the defects and the formation of amorphous carbon during annealing in inert atmosphere, hence ameliorating the wall integrity of the annealed SWCNTs. It is worth noting that although EMI SE increased significantly after annealing, the long-SWCNTs composites without annealing performed much better, which verified that the EMI SE is more closely related to the aspect ratio other than wall integrity.

**Figure 4:**
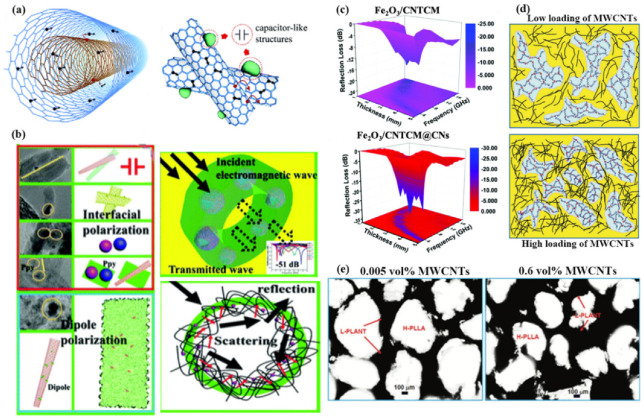
(**a**) Schematic of electron transport in MWCNT and ZnO@CNTs. Reproduced with permission from Lu et al. [8], Multi-wall carbon nanotubes decorated with ZnO nanocrystals: mild solution-process synthesis and highly efficient microwave absorption properties at elevated temperature; published by Royal Society of Chemistry, 2014. (**b**) Schematic of the microwave loss of the Fe_2_O_3_/CNTCM@CN composites. (**c**) Microwave reflection losses with the 3D map images of Fe_2_O_3_/CNTCM and Fe_2_O_3_/CNTCM@CNs. Reproduced with permission from Yu et al. [69], Improved microwave absorption performance of a multi-dimensional Fe_2_O_3_/CNTCM@CN assembly achieved by enhanced dielectric relaxation; published by Elsevier, 2020. (**d**) Schematic illustrations of the MWCNT redistribution in PLLA/PCL/MWCNT (PLLA/PCL 60/40) nanocomposites with low and high loadings of MWCNTs with 20 wt% PDLA in the PLLA phase. Reproduced with permission from Zhang et al. [70], Morphological regulation improved electrical conductivity and electromagnetic interference shielding in poly(l-lactide)/poly(ε-caprolactone)/carbon nanotube nanocomposites via constructing stereocomplex crystallites; published by Royal Society of Chemistry, 2017. (**e**) OM images of the S-PLLA/MWCNT composites with MWCNT contents of 0.05 vol% and 0.6 vol%. Reproduced with permission from Zhang et al. [71], Ultralow percolation threshold and enhanced electromagnetic interference shielding in poly(l-lactide)/multi-walled carbon nanotube nanocomposites with electrically conductive segregated networks; published by Royal Society of Chemistry, 2017.

Various strategies have been exploited to modify CNTs to enhance the impedance matching and microwave dissipation. Yu et al. [69] assembled 0D Fe_2_O_3_ nanoparticles, 1D CNTs, and 2D N-doped carbon layers, obtaining Fe_2_O_3_/CNTCM@CN-2 magnetic-dielectric composite microspheres. The capacitor-like structure provides additional interfacial polarization (Figure 4b). Although the poor magnetic dissipation behavior of this hybrid still exists after adding magnetic particles, the over-encapsulation of N-doped carbon layers plays a dominant role in adjusting microwave absorption performance. The decreased dielectric loss and increased magnetic loss lead to better impedance matching (Figure 4c). In addition, semiconductors, such as ZnO and CdS, are desired for widening the absorption frequency bandwidth [8,48]. The capacitor-like structures strengthen the polarization loss by the interface between the CdS/ZnO and CNTs (Figure 4a). It is also crucial in judiciously engineering the nanostructures of the CNTs-based composites. Zhang et al. [70] demonstrated a morphological control of the MWCNT conductive network with poly(L-lactide) (PLLA) and poly(ε-caprolactone) (PCL) blends during the formation of stereo complex crystallites (SCs) under melt processing. The co-continuous 1D morphology, as well as the sufficient contents of the MWCNTs, constructs the conductive network, leading to the enhancement of the dielectric loss (Figure 4d). However, the mechanical strength of the composites is inferior because of the low compatibility between the aforementioned polymers. Later, Zhang and co-workers [71] solved the mechanical problems by constructing a segregated structure in two polymers with a similar molecular structure but different viscosities (Figure 4e). They built segregated PLLA/MWCNT (S-PLLA/MWCNT) using two PLLAs with different viscosities and compared their EMI SE with a random distributed MWCNTs (R-PLLA/MWCNTs). The results showed that S-PLLA/MWCNT composites had higher conductivity at ultralow percolation threshold than that of R-PLLA/MWCNT, which was ascribed to the continuous and dense MWCNT in S-PLLA/MWCNT. According to the relevant reports, it can be verified that the modification of N elements, magnetic particle, and semiconductors is favorable for the impedance matching, as well as the increment of the effective absorption bandwidth. Moreover, the well-dispersion of the CNTs and the design of the structure of the absorber can enhance the dielectric loss and the mechanical strength, prolonging the service life.

Carbon fiber, another fascinating 1D dielectric-loss material with a carbon content of more than 95%, provides advantages such as being lightweight, flexible, and having high electrical conductivity, as well as good absorbency of EM wave [72,73]. In comparison with CNTs, carbon nanofiber features a fibrous structure, which has high strength and modulus along the fiber axis, due to the orientation of the fiber axis along the growth of graphite crystallite. Moreover, it is a well-accepted method used to entangle long carbon fibers together to form a certain structure, facilitating the construction of an applicable non-woven textile [49]. Furthermore, the irregular crossed section of carbon nanofibers can promote the absorption of the microwave through the multiple reflection inside the absorbing body. However, high resistivity and brittleness in the nature of carbon fiber limits its application in the EMI shielding composites [74]. Lei et al. [75] enhanced the electrical conductivity and EMI shielding performance of CNT through coating the nano-scale Au particles. Meanwhile, polydimethylsiloxane (PDMS) polymer was infiltrated into Au@CNT/sodium alginate sponge skeleton to boost its flexible property. After 10 elastic tensile strain, the conductivity of Au@CNT/sodium alginate/PDMS flexible composites remained unchanged. Chen and co-workers [9] simulated the anisotropy of the carbon fiber by depicting carbon parallel to the X- and Y-axis, demonstrating that the resistance is mainly dominated by SiC contents. With the increment of SiC, pores on the surface of C_f_/SiC were gradually blocked by the SiC matrix, which declined the EM wave impedance matching between the air and the sample. The mechanical strength was improved with the enhanced contents of SiC from 21.5 to 42.2 vol%, owing to the loading transfer to carbon fiber. Unexpectedly, the EMI SE decreased from 42.7 to 31.4 dB over the frequency range 8.2–12.4 GHz due to the decrease in electrical conductivity. Moreover, the reflection loss remained almost unchanged and was kept at around 10 dB with the variation of SiC contents, suggesting that the mechanical properties and EMI SE can be facilely modulated to meet the requirements [75]. Thus, the EM wave absorber and shield with multiple components can achieve decent absorption capability due to the synergistic effects of multiple loss mechanism. Moreover, the 2D carbon fiber composites possess ultra-thin and superlight property, which is suitable for the further applications for wearable devices and portable electronics.

#### 3.1.2. 2D Nanomaterials

Graphene, a typical two-dimensional (2D) carbon material, has been demonstrated as an emerging and promising material in the area of EMI shielding because of its remarkable properties, such as excellent mechanical strength, large surface area, efficient charge mobility [76], and decent thermal conductivity [77,78,79]. Apart from graphene film and sheets [80], various graphene-based composites, such as graphene/polymer composites [52] and graphene/metal oxide composites [81], have been widely reported as EMI shielding materials because of their low density, flexibility, and absorption dominant shielding. Kumar et al. [82] demonstrated that the large-area graphene film possesses higher electrical conductivity and thermal conductivity, and thus exhibits superior EMI shielding performance. Wan et al. conducted a further investigation on the size of GO to EMI shielding effectiveness [53]. The large-sized reduced graphene oxide (LG) paper (8.4 µm) has the better EMI shielding capability (44.7 dB) compared with middle-sized reduced graphene oxide (MG) paper and small-sized reduced graphene oxide (SG) paper. The high SE is ascribed to fewer defects, more conjugated carbon domains, and better alignment of the LG, which resulted in higher carrier mobility and a continuous conductive network. As a result, LG has been proved to be the optimum graphene sheets for further study. In addition, the increased electrical conductivity and decreased skin depth are the reasons why the iodine-doped LG significantly improves the EMI SE. The works mentioned above provide a guideline of hybridizing graphene-derived material in the field of EM functional materials.

Taking the environmental irradiation into account, the conductive materials are anticipated to absorb as much of the incident wave as possible. Ideally, the EM wave absorption materials should perform outstanding absorption capability and less reflection and transmission to avoid the secondary pollution. Hen et al. [83] introduced the porous structure into graphene film via the hydrazine-foaming method. Although graphene film has higher electrical conductivity, the foam structure weakens the back reflection and scattering between the air and graphene layers, thereby promoting the absorption of the radiation energy. The work demonstrated that the enhancement of about 30% in EMI shielding could be achieved by graphene foam with lower electrical conductivity (~ 3.1 ± 0.8 S·cm^−1^) due to the presence of microcellular structure.

**Figure 5:**
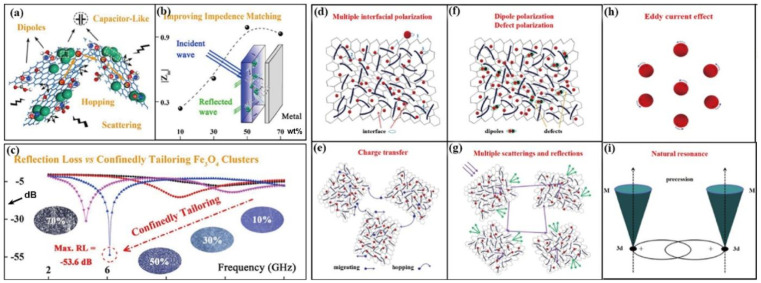
(**a**) EM wave response, (**b**) |*Z_in_*| and (**c**) RL of the Fe_2_O_3_-GN. Reproduced with permission from Wang et al. [84], Confinedly tailoring Fe_3_O_4_ clusters-NG to tune electromagnetic parameters and microwave absorption with broadened bandwidth; published by Elsevier, 2018. Schematic of (**d**) multiple polarization, (**e**) charge transfer, (**f**) dipole and defects polarization, (**g**) multiple scatterings and reflections, (**h**) eddy current, and (**i**) natural resonance of the RGO/thorns-like PANI/α-Fe_2_O_3_@SiO_2_ nanocomposites. Reproduced with permission from Zhang et al. [85], Synthesis of graphene/thorns-like polyaniline/α-Fe_2_O_3_@SiO_2_ nanocomposites for lightweight and highly efficient electromagnetic wave absorber; published by Elsevier, 2018.

Graphene, with abundant groups and defects, can be facilely modified. Some dielectric and magnetic particles can be loaded on the graphene. Wang and co-workers [84] implanted magnetic Fe_2_O_3_ clusters on the N-doped graphene (GN), improving the microwave absorption up to −53.6 dB. Zhang et al. [85] grew the dielectric medium, polyaniline (PANI) and SiO_2,_ and the magnetic medium on the surface of graphene oxide. The polarization (i.e., interfacial polarization and the Debye dipolar relaxation) contributes to the impedance matching by introducing dielectric-magnetic medium, thus enhancing the electromagnetic wave absorption performance (Figure 5). The dielectric-magnetic synergy can definitely modulate EM response of the materials. Furthermore, the construction of nanomaterials with a controllable electro-magnetic gradient also induces more striking results since it can lead to interfacial polarization and relaxation polarization owing to its novel structure. Liu et al. [86] adjusted the arrangement and content of the Fe_3_O_4_@rGO and the MWCNT nanofiller in waterborne polyurethane (WPU). The composites present a positive electrical conductivity gradient and negative magnetic gradient. Therefore, the incident wave undergoes a particular “absorption-reflection-reabsorption” process and polarization loss between impedance matching layer and high conductive layer, leading to low RL value and high electromagnetic shielding. As for the graphene-based absorber, large-surface graphene endows a higher carrier mobility and a continuous conductive network. The impedance matching can be optimized via magnetic particle dopant and structure designing. The induced polarization relaxation process plays a dominate role in the dissipation of the electromagnetic waves.

#### 3.1.3. 3D Nanomaterials

3D continuous porous structures provide merits such as large specific surface area and low gravimetric density; and play a dominate role in various fields, such as energy conversion [87], energy storage [88], microwave absorption [89] etc. Due to the giant 3D cross-linked network and hierarchical architectures, the 3D nanostructure exhibits tunable complex permittivity and conductivity, which contributes to remarkable conduction loss and polarization loss [89]. Moreover, porous materials usually exhibit better impedance compatibility than non-porous material.

Conductive graphene/polymer foam with lower density (0.3 g·cm^−3^) was reported by Ling et al. [55]. The foaming process significantly elevated the specific EMI SE from 17 to 44 dB·g^−1^·cm^−3^ owing to the accumulation and orientation of graphene on the cell wall during the cell growth. About 90.6 to 98.9% of electromagnetic energy was absorbed by the microcellular. Nevertheless, high loading of carbon nanofillers is usually required to improve the EMI SE, which unavoidably leads to a complicated manipulation process and obtains a fragile composite. Zeng et al. [56] prepared a lightweight and anisotropic MWCNT/WPU composite. They studied the structure-property relationship between the different porous orientation and shielding capability. The results implied that the high conductive filler is beneficial for the EMI shielding performance. To solve the problem, Chen et al. [57] built a 3D carbon nanotube sponge as the 3D reinforcement and conductive network, which achieved a remarkable conductivity of 148 S·m^−1^ and a prominent EMI SE of around 33 dB with only 0.66 wt% loading of CNT sponge at an ultrathin thickness (2 mm). Li et al. [90] fabricated a core-shell graphene-bridge hollow MXenes spheres (RGO/Ti_3_C_2_T_x_) 3D foam with high EM absorption capability. The unique heterostructure is formed by grafting RGO on the surface of Ti_3_C_2_T_x_. The defects, such as the boundaries, stacking faults, and surface functional groups can enhance the polarization loss. Foams, aerogels, and sponges with numerous internal pores, enhance the multiple reflection loss, decline the fillers contents, and widen the frequency bandwidth. The 3D porous structure can prevent the EM waves from reflecting or penetrating before being absorbed.

In summary, the decent conductive and dielectric properties of the carbon-based composites contribute to good conductive loss and dielectric loss owing to their high degree of graphitization. Moreover, the design of the carbon-based absorbers with hierarchical architectures, high surface area, and heterogenous structures can facilitate the interfacial polarization loss. In addition, the development of carbon/magnetic hybrids is one of the most effective strategies to boost the impedance match due to the synergetic effect of magnetic loss and dielectric loss.

### 3.2. Other Conductive Materials

#### 3.2.1. MXenes-Based Materials

MXenes, a novel family of 2D early transition carbides/nitrides/carbonitrides, are firstly prepared in 2011 by selectively etching certain components from their layered MAX phase [91]. Due to the large surface area, outstanding electrochemical properties, decent mechanical strength and metallic conductivity [92,93,94] MXenes has been regarded as an alternative for superior EMI shielding and absorption materials.

It has been proved that the EMI shielding effectiveness and microwave absorption of the Ti_3_C_2_T_x_ is higher than those of precursor TiAlC_2_ [95,96]. The enhanced reflection loss value is predominantly ascribed to the presence of the surface functional groups, defects, and multiple interfaces in-between the layers, which gives rise to polarization loss, and thus significantly contributes to dielectric loss [97,98]. In addition, a different layer number has caused considerable influence on the conductive and dielectric capability. It has been demonstrated that the conductivity of the single-layered Ti_3_C_2_T_x_ is comparable to graphene and is two orders of magnitude higher than MoS_2_ [99]. Furthermore, Lipatov et al. [100] confirmed that the conductivity of single flake is one order of magnitude higher than that of the bulk Ti_3_C_2_T_x_. Consequently, the imaginary part of the relative permittivity of the single-layered and few-layered Ti_3_C_2_T_x_ is higher than that of the multi-layered Ti_3_C_2_T_x_, and different conduction loss capability can be anticipated.

**Figure 6:**
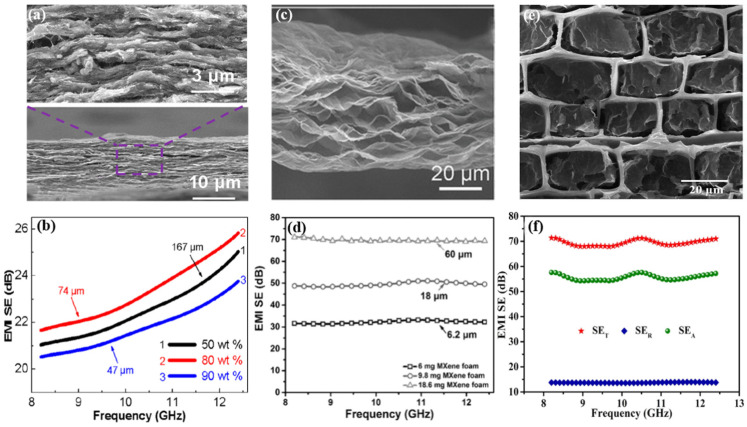
(**a**) SEM images of d-Ti_3_C_2_T_x_/CNF composite paper with different content of Ti_3_C_2_T_x_, (**b**) Effect of d-Ti_3_C_2_T_x_ content on the EMI SE of the d-Ti_3_C_2_T_x_/CNF composite paper sheets with different d-Ti_3_C_2_T_x_ contents and different thicknesses in the X-band region. Reproduced with permission from Cao et al. [102], Binary strengthening and toughening of MXene/cellulose nanofiber composite paper with nacre-inspired structure and superior electromagnetic interference shielding properties; published by American Chemical Society, 2018. (**c**) SEMs image of MXene foam, (**d**) EMI SE of the MXene foams with different thicknesses. Reproduced with permission from Liu et al. [11], Hydrophobic, flexible, and lightweight MXene foams for high-performance electromagnetic-interference shielding; published by John Wiley & Sons, 2017. (**e**) SEMs image of MXene aerogel/WPC composites, (**f**) EMI SE of MXene aerogel/WPC composites. Reproduced with permission from Liang et al. [103], Ultra-light MXene aerogel/wood-derived porous carbon composites with wall-like “mortar/brick” structures for electromagnetic interference shielding; published by Elsevier, 2020.

Most MXenes-based absorbers use the single-layered and few-layered Ti_3_C_2_T_x_ with soft polymer matrices (cellulose [101,102] and elastomer [93,94]). Cao et al. [101] fabricated ultrathin and highly flexible MXenes/cellulose nanofiber (CNF) paper with nacre-like lamellar structure (Figure 6a). The paper not only ensured an excellent EMI shielding efficiency (up to 25.8 dB) and high electrical conductivity (up to 739.4 S·m^−1^) at a thickness of only 47 µm but also presented excellent tensile strength (up to 135.4 MPa) and fracture strain (up to 16.7%) (Figure 6b). It is noteworthy that CNF with a 1D structure acts as the binding agent to connect delaminated Ti_3_C_2_T_x_ (d- Ti_3_C_2_T_x_) with a 2D structure to form the anisotropic interconnection networks with less insulating contacts leading to the enhanced mechanical strength. The nacre-like lamellar structure provides more interface contact for the incident wave, which facilitates the multiple internal reflection. In pursuit of further enhancing the EMI shielding performance with decent mechanical strength, Cao’s group prepared an ultrathin and flexible carbon nanotubes/MXenes/cellulose composite paper with gradient and sandwich structure [102]. To analyze the influence of the sandwich structure and gradient structure on EMI SE enhancement, the paper was prepared with a symmetric layered structure and two-layered CM composite with various Ti_3_C_2_ contents. The results demonstrated that the sandwich structure is more favorable for the improvement in EMI SE. The gradient structure showed a distinct effect on the value of SE_A_ and SE_R_. As a result, the sandwich structure is a good candidate for preparing layered composites.

Porous foams and aerogels are highly suitable because of their low density and enormous interfaces. Liu et al. [11] employed LiF/HCl-etching treatment of Ti_3_C_2_T_x_ to prepare a hydrophobic MXene foam (Figure 6c). Due to the high electrical conductivity and the increased thickness, the ultimate EMI SE was enhanced and reached up to ~70 dB, much higher than that of the MXene film (53 dB) (Figure 6d). Recently, ultralight MXene aerogel/wood-derived porous carbon composites with “mortar/brick” structures (Figure 6e) was reported [103]. The EMI SE reached up to 71.3 dB (Figure 6f). The unique structure of the hybrid composite greatly stabilizes the MXene aerogel networks, as well as prolonging the transmission paths of the electromagnetic waves and dissipating the incident waves into heat energy. 1D continuous carbon nanostructure speeds up the electron transport and enhances the impedance matching between the composites and air. Moreover, MXene aerogel network inside of the cell of the wood-derived porous carbon provides more interfaces, which induces the interfacial polarization. The multi-scattering mechanism in the composites boosts the absorption performance. Moreover, the heat treatment of MXene decreases the hydroxyl groups, meanwhile forming the amorphous carbon without destruction of the 2D structure, which has a positive effect on conductive loss due to the increased electronic conductivity [104]. Furthermore, the anatase and rutile TiO_2_ nanocrystals and amorphous carbon optimizes the impedance match with free space and generated dielectric dipole interaction at multi-interfaces [105].

Those works provide the guideline for the MXene-derived absorber with green and sustainable lignocellulose. The film and the aerogel in range of the micro-nano scale can further assemble with other dielectric or magnetic layers to obtain hierarchical structure to elevate the EM absorption capability.

#### 3.2.2. Conductive Polymer

Conductive polymer, a type of organic material with highly π-conjugated polymeric chains, such as polyaniline (PANI), polypyrrole (PPy), polyacetylene (Pan), etc., has attracted much attention for EMI shielding application, as it provides the ability to efficiently switch between the redox states, excellent ion-exchange property, and controllable electrical conductivity [106]. In addition, the organic conductive polymer also undergoes a swelling, contraction, and cracking process so that it can influence their mechanical and electrical properties in turn. The inferior position can be improved by hybridizing other materials with merits of high mechanical strength, conductive, and magnetic property, such as cellulose, carbon nanotube, graphene, and magnetic medium [107,108]. A cellulose skeleton with 3D structure was fabricated with PANI conducting polymer cladding on its surface with high EM absorption characteristic and thermal insulation property [109]. Due to the large surface area, the large contact area will be exposed between material and air, leading to a space charge polarization, boosting the microwave to be dissipated. While PANI has a higher infrared reflectance and a lower microwave reflectance, the hybrid aerogel exhibits strong microwave loss and is compatible with thermal insulation.

Wu et al. [12] developed graphene foam (GF)/poly(3,4-ethylenedioxythiophene): poly(styrenesulfonate) (PEDOT: PSS) composites through drop coating on cellular-structured GFs. The results indicated that the electrical conductivity of the composites was raised after introducing the GFs to the PEDOT: PSS. The EMI SE of GF/ (PEDOT: PSS) could be over 90 dB. In regard to the conductive polymer, the heterogeneity not only enhances the dielectric permittivity but also leads to an unordered motion of charge carrier along the chain of the conductive polymer, which improves microwave absorption.

### 3.3. Magnetic Materials

Magnetic materials are good candidates for magnetic attenuative sources in EMI shielding materials due to the high magnetic saturation and low coercivity [110,111]. High magnetic permeability endows the magnetic components with high additional magnetic loss besides the improvement of the impedance match, leading to an extended absorption bandwidth [112]. At this point, various magnetic absorbents such as magnetic metals and alloys, ferrites, as well as their composites have been studied to enhance the magnetic permeability and adjust dielectric permittivity for absorption enhancement [113], as displayed in Table 2.

**Figure 7:**
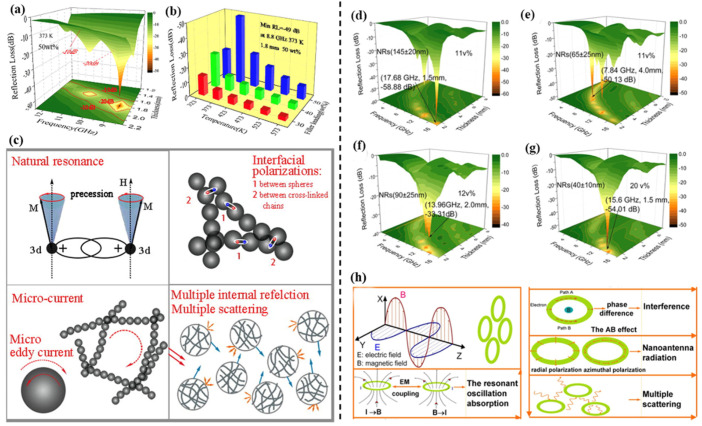
(**a**) 3D plot of RL versus frequency and thickness. (**b**) Maximum RL versus temperature and the filler loading. (**c**) Schematic for RL mechanism of the nickel nanochains. Reproduced with permission from Liu et al. [114], Electromagnetic property and tunable microwave absorption of 3D nets from nickel chains at elevated temperature; published by American Chemical Society, 2016. (**d**–**g**) RL versus volume fractions and thickness. (**h**) Contribution of the ring-like configuration to microwave absorption. Reproduced with permission from Tong et al. [13], Tunable dielectric properties and excellent microwave absorbing properties of elliptical Fe_3_O_4_ nanorings; published by American Institute of Physics, 2016.

Liu et al. [116] fabricated 1D nickel nanochains with large saturation magnetization and wide resonance. The optimum RL reached −50 dB, even at elevated temperatures (Figure 7a,b). There is a synergistic effect of natural resonance, micro eddy current, and interfacial polarization (Figure 7c). Interestingly, 2D Fe microplates have large shape anisotropy, which can break Snoek’s limit [117]. The maximum RL of the single-component Fe microplates is −43.4 dB, equaling to those of the multiple-components absorbers. For magnetic alloy materials, the electron transfer and spin polarization between different metal atoms in the magnetic alloy materials are helpful for the further enhancement of the absorbing capacity [127]. Cheng et al. [128] fabricated the FeCo alloys in which the magnetic loss of the alloys is mainly arisen from natural resonance and exchange resonance.

Ferrite can also exhibit excellent EM absorption properties, and present higher resistivity (10^8^–10^12^ Ω cm) than other magnetic metals or alloys. In this regard, it can avoid the skin effect at high frequency [114]. Tong et al. [13] investigated the structure-property relationship between the microwave absorption and the size of elliptical Fe_3_O_4_ nanorings (NRs). Because the exciting electron generated by induced electromotive force and current significantly enhanced the orientation/interface polarization in the ring cavity, the dielectric loss of elliptical Fe_3_O_4_ nanorings depended on the long axis (Figure 7d–g), instead of the defects, aspect ratio, and specific surface area [129]. Furthermore, the unique ring-like configuration of the Fe_3_O_4_ NRs dramatically enhanced the multi-scattering, oscillation resonance absorption, microantenna radiation, and interference loss, which contributes to attenuate the energy of the EM wave (Figure 7h). It is difficult for single phase ferrite to satisfy the demands of the EM absorber in the frequency range of 2–18 GHz. To cope with the problem, Huang et al. [115] prepared hollow BaFe_12_O_19_/CoFe_2_O_4_ microrods with excellent input impedance matching and efficient microwave absorption bandwidth. The superior microwave absorption property is attributed to the high saturation magnetization of the soft phase and high coercivity of the hard phase.

In summary, the excellent magnetic properties based on the magnetic materials originates from the eddy current loss, natural resonances, and magnetic losses. Moreover, the construction of the structures (i.e., core-shell structure, chain, elliptical nanoring) can also inherit a certain of reflection loss. However, the higher density of the aforementioned magnetic materials restricts them the possibility of EM absorption application.

### 3.4. Metal-Organic Framework (MOF)-Derived Materials

The shielding efficiency and EM absorption capacity of the materials depend on the synergistic effects of electrical conductivity and the magnetism strength. Thus, it is feasible to combine the dielectric matrix and magnetic materials to attenuate the EM wave with a moderate permittivity and permeability [130,131,132]. Some conventional composites have been fabricated and have served as absorption-dominated shielding materials, such as RGO/PANI/Cu_2_O [133], NiFe_2_O_4_/rGO [134], Co-C-MWCNTs [135], CoFe_2_O_4_/C/PANI [136], and so on. Nevertheless, there is still difficulty in achieving excellent EM absorption capacity. The bottleneck is how to prepare the outstanding EM wave absorption material by optimizing the composition and configuration in a simple way.

Metal-organic framework (MOF) materials, which are constructed from metal ion/clusters (joint) and organic ligands (linker), stand for a new class of crystalline porous material with periodic network structure [137,138,139]. Since the concept of MOF was first proposed by Yaghi’s group in 1995 [140], MOF materials have attracted enormous research interests and have been playing an important role in the development of new materials in gas absorption and separation [141], catalysis [142,143], energy storage [144,145], and drug delivery [146], due to their high porosity and large surface area with well-dispersed features. Recently, MOF-derived composites have been demonstrated to be promising candidates for wave absorbers owing to its rich magnetic metal nodes and highly conductive carbon after thermal annealing [19,121,122,147], as displayed in Table 2. A porous structure with large surface areas and sufficient porosity leads to the lowering of the permittivity [123,148]. On the one hand, the massive continuous hollows serve as polarization centers, further enhancing the dielectric loss ability [149,150]. On the other hand, graphite-like crystal can be prepared with metal catalysis during the pyrolysis, thereby enhancing the dielectric loss ability of the material. Meanwhile, the magnetic ions (Fe, Co, Ni) can be converted into the magnetic metals, enhancing the magnetic loss. Furthermore, the structural and compositional tunability endows MOF as an excellent choice in EMI shielding field.

**Figure 8:**
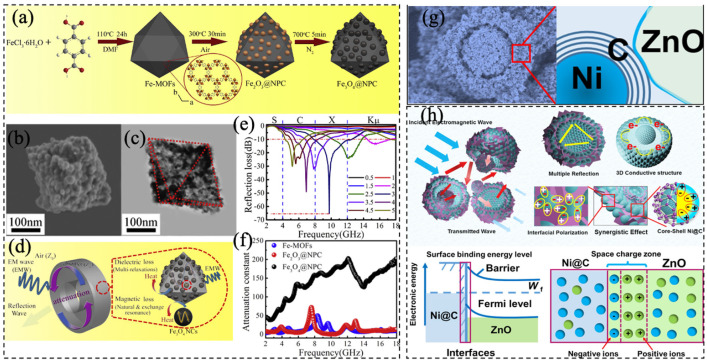
(**a**) The schematic illustration of the Fe_3_O_4_@NPC composites formation process. (**b**) SEM images, (**c**) low magnification TEM image of Fe_3_O_4_@NPC composites. (**d**) Schematic of the electromagnetic wave absorption mechanism. Frequency-dependent (**e**) attenuation constant and (**f**) RL. Reproduced with permission from Xiang et al. [17], Enhanced electromagnetic wave absorption of nanoporous Fe3O4@carbon composites derived from metal-organic frameworks; published by Elsevier, 2019. (**g**) SEM image and the illustration of the yolk-shell Ni@C@ZnO microspheres. (**h**) The microwave absorption mechanism. Reproduced with permission from Wang et al. [122], MOF-derived yolk-shell Ni@C@ZnO Schottky contact structure for enhanced microwave absorption; published by Elsevier, 2020.

Xiang et al. [17] prepared the nanoporous Fe_3_O_4_@NPC composites from Fe-MOF (Figure 8a). After annealing treatment, its structure remained the same as before (Figure 8b,c). The RL value and the attenuation constant of the composite increased with the increased calcination temperature (Figure 8e,f), which was attributed to the synergistic effect of the dielectric loss and the magnetic loss. Furthermore, the heterointerfaces between Fe_3_O_4_ and the carbon medium boost the interfacial polarization and associated relaxation, thereby enhancing the interior multi-refection. (Figure 8d). It is also favorable to a construct yolk-shell structure with a distinct layered configuration for EM wave absorption. Wang et al. [124] designed a hierarchical multi-interfacial Ni@C@ZnO microsphere after annealing the bimetallic Ni-Zn-MOF, which possessed the “Schottky contact barrier” in the MOF-derived Ni@C@ZnO ternary composites (Figure 8g). The core-shell Ni@C micro-units and ZnO flakes were assembled to form a unique yolk-shell microsphere. The Schottky contact barrier at the Ni@C-ZnO interface intensified the interfacial polarization. By taking advantage of the magnetic-dielectric synergistic effect, the optimal bimetallic Ni@C@ZnO composites exhibited outstanding EM wave absorption and showed the maximum of RL at 2.5 mm (Figure 8h). The effective wave absorption bandwidth (RL ≤ 10 dB) covered 4.1 GHz with a low mass ratio of 25%.

Generally speaking, similar to conventional magnetic carbon hybrid materials, the absorption capability of MOF is arisen from the impedance matching and the dual-loss mechanism. Both the conductive medium with superior dielectric properties and the magnetic medium with the good magnetic properties possess excellent dielectric loss and magnetic loss, thus enhancing the impedance matching and the EM absorption. Moreover, the heterogeneous interfaces between conductive materials and magnetic materials promote the interfacial polarization, increase the scattering of waves, and enhance the microwave attenuation.

## 4. Conclusions and Outlook

In summary, several kinds of nanomaterials with superior EM wave absorption capabilities have been systematically reviewed herein along with the critical comments on the EM wave absorbing behaviors of various composites from the loss mechanism and assembling configuration perspectives. Besides, the conduction loss from the conductive materials and magnetic loss from the magnetic components, the highly porous structures with boosted surface area promote the surface scattering and multiple reflection, while the core-shell configurations enhance the interfacial polarizations. Additionally, the abundant heteroatomic dopants introduce dipolar polarization from a view of atoms and surrounding electronic clouds. A synergistic optimization of the aforementioned materials can significantly widen the effective bandwidth, facilitate the absorbing/shielding capability, lower the matching thickness, and minimize the filling ratio in the paraffin matrix. However, there is limited research on the fabrication of multicomponent hybrid EM wave absorber. It is imperative to develop the wave absorbers with tailored and well-defined structures such as uniform pore size and hierarchical architecture at multiple levels through the structure-property relationship from a fundamental point of view. It is highly desirable that the absorbers can offer a myriad of merits such as being light-weight, flexible, tough, environmentally friendly, and low-cost, which is greatly important for future practical applications especially in portable electronic devices.

## Figures and Tables

**Table 1:** The EM loss mechanisms of different dimensional carbon nanomaterials.

Dimension	Carbon Materials	Microstructure	Electromagnetic Loss Mechanism	Ref.
1D carbonaceous materials	CNT/cellulose	Film	Dielectric loss, multiple reflection	[47]
	Cds-CNT	Core-shell nanowire	Dielectric loss, interfacial polarization	[48]
	ZnO@MWCNT	Hybrid	Capacitor-like structure, interfacial polarization, impedance matching, dielectric loss	[8]
	Ag nanowire/Carbon fiber	Fabric	Conduction loss, multiple reflection and scattering	[49]
	Carbon fiber/SiC	Hybrid	Conduction loss, reflection at various surface or interface	[9]
	Carbon fiber/Si_3_N_4_	Hybrid	Electronic relax polarization, conductive loss, impedance	[50]
	Carbon fiber	Hollow	Hollow structure accelerates the increasing rate part while lowering that of the imaginary part	[51]
2D carbonaceous materials	rGO/cellulose	Film	Multiple reflection loss, dielectric loss	[52]
	rGO	Film	Dipole polarization originate from few defects, better alignment of the large area	[53]
	rGO	Nanosheet	Dielectric loss, impedance matching	[54]
3D carbonaceous materials	Polyetherimide/rGO	Sponge	Multiple interface reflection, dielectric loss	[55]
	MWCNT/WPU	Foam	Multiple reflection loss at various surface and interface, conduction loss, dielectric loss	[56]
	Epoxy/carbon nanotube	Sponge	Conduction loss, abundant interfaces that multiply the reflection	[57]
	MWCNT/Graphene	Foam	High loss multilevel network architecture	[58]
	PANI/GO	Aerogel	Impedance matching, multiple reflection, electron polarization	[59]

**Table 2:** The EM loss mechanism of magnetic nanomaterials.

Classify	Magnetic Materials	Microstructure	Electromagnetic Loss Mechanism	Ref.
Ferrite	Fe_3_O_4_	Nanocrystal	Natural resonance	[114]
	Fe_3_O_4_	Nanoring	Orientation/interface polarization, dielectric loss, oscillation resonance absorption	[13]
	BaFe_12_O_19_/CoFe_2_O_4_	Hollow microrod	High saturation magnetization of the soft phase and high coercivity of the hard phase	[115]
Magnetic metal	Ni	Chain	Natural resonance, micro eddy current, interfacial polarization	[116]
	Fe	Microplates	Magnetic loss, conduction loss	[117]
	Ni	Nanoparticle	More interfacial polarization	[118]
Magnetic alloy	Fe_7_Co_3_	Layer-like	Magnetic loss is domain, impedance matching	[119]
	CoNi	Flower like	Interfacial magnetic dipole interaction, multiple scattering in the space woven	[120]
	Co_20_Ni_80_	Urchin-like	Eddy-current loss, magnetic hysteresis loss	[16]
MOF-derived material	Co/C	Porous	Synthesized effects between the multiple components and highly porous structure, dielectric loss, magnetic loss	[19]
	Fe-Co/graphene	Dodecahedrons	Dielectric loss, magnetic loss	[121]
	Ni/C	Hollow	Electronic dipole polarization, multiple refection, interfacial polarization, conduction loss	[122]
	Co-C/MWCNT	Hollow	Orientation-enhanced dielectric and magnetic loss, impedance matching	[123]
	Ni@C@ZnO	Yolk-shell	Schottky contact barrier intensifies the interfacial polarization, magnetic-dielectric synergistic effect	[124]
	CoFe@carbon	Fiber	Abundant interfacial polarization, multi-scattering, magnetic loss	[125]
	Co@NC@rGO	Nanosheets	Magnetic loss, interfacial polarization	[126]

## Data Availability

Data is contained within the article.
